# Whole Genome Sequencing Based Taxonomic Classification, and Comparative Genomic Analysis of Potentially Human Pathogenic *Enterobacter* spp. Isolated from Chlorinated Wastewater in the North West Province, South Africa

**DOI:** 10.3390/microorganisms9091928

**Published:** 2021-09-10

**Authors:** Tawanda E. Maguvu, Cornelius C. Bezuidenhout

**Affiliations:** Unit for Environmental Sciences and Management-Microbiology, North-West University, Private Bag X6001, Potchefstroom 2520, South Africa; carlos.bezuidenhout@nwu.ac.za

**Keywords:** *Enterobacter*, comparative genomics, pan-genome analysis, taxonomic classification

## Abstract

Comparative genomics, in particular, pan-genome analysis, provides an in-depth understanding of the genetic variability and dynamics of a bacterial species. Coupled with whole-genome-based taxonomic analysis, these approaches can help to provide comprehensive, detailed insights into a bacterial species. Here, we report whole-genome-based taxonomic classification and comparative genomic analysis of potential human pathogenic *Enterobacter hormaechei* subsp. *hoffmannii* isolated from chlorinated wastewater. Genome Blast Distance Phylogeny (GBDP), digital DNA-DNA hybridization (dDDH), and average nucleotide identity (ANI) confirmed the identity of the isolates. The algorithm PathogenFinder predicted the isolates to be human pathogens with a probability of greater than 0.78. The potential pathogenic nature of the isolates was supported by the presence of biosynthetic gene clusters (BGCs), aerobactin, and aryl polyenes (APEs), which are known to be associated with pathogenic/virulent strains. Moreover, analysis of the genome sequences of the isolates reflected the presence of an arsenal of virulence factors and antibiotic resistance genes that augment the predictions of the algorithm PathogenFinder. The study comprehensively elucidated the genomic features of pathogenic *Enterobacter* isolates from wastewaters, highlighting the role of wastewaters in the dissemination of pathogenic microbes, and the need for monitoring the effectiveness of the wastewater treatment process.

## 1. Introduction

The genus Enterobacter comprises common human pathogens, with the Enterobacter cloacae complex (ECC) species (Enterobacter cloacae subsp. cloacae, Enterobacter asburiae, Enterobacter kobei, Enterobacter hormaechei, Enterobacter ludwigii among others) being notoriously found in hospitals, causing a wide range of infections such as lower respiratory tract infections, urinary tract infections, bacteremia, and meningitis [1,2,3]. The clinical significance of the ECC strains is aided by the fact that most of the strains carry multiple antibiotic resistance genes [4,5]. Although the ECC strains are of clinical significance, global surveillance and characterization of the virulence factors associated with these species are still limited [6].

Precise species and subspecies assignation of bacterial isolates lays a foundation for understanding the epidemiology, pathogenesis, and microbiological features of bacteria, allowing for better global surveillance and comparative genomics. Taxonomic classification of the genus *Enterobacter* is complicated; recently, Wu et al. (2020) updated the taxonomy of *Enterobacter* species and subspecies based on whole-genome taxonomic analysis [7]. The complexity resulted from the fact that classification in the genus *Enterobacter* had been defined based on low-resolution analytical methods, and is therefore in need of careful re-examination [8,9,10]. As shown with the current severe acute respiratory syndrome coronavirus 2 (SARS-CoV-2), proper identification of strains and variants is critical, as they have important implications for diagnosis, treatment, prognosis, and prevention. Whole-genome sequences have greatly improved the identification of species, as they elucidate functional profiles of taxonomic groups and easily resolve ambiguities in the phylogeny of higher taxa, which would have been difficult through traditional approaches [11,12]. 

In the current study, we present whole-genome-based taxonomic classification and comparative genomic analysis of potential human pathogenic *E. hormaechei* subsp. *hoffmannii* (designated as isolates S4 and S5) isolated from chlorinated wastewater. This study aids with the global baseline surveillance of ESKAPE pathogens and provides useful resources (genome sequences), which further helps understanding of the global dynamics of an important bacterial species, *Enterobacter*. More importantly, the taxonomic classification analysis from this study supports a recent report by Wu et al. (2020) that *E. cloacae* subsp. *dissolvens* and *E. hormaechei* subsp. *hoffmannii* are species rather than subspecies; *Enterobacter xiangfangensis*, *E. hormaechei* subsp. *oharae*, and *E. hormaechei* subsp. *steigerwaltii* are the same species. Thus, it is important to apply whole-genome-based taxonomic classification, particularly when dealing with isolates of clinical relevance, since their proper identification is critical for proper diagnosis, treatment, prognosis, and prevention.

## 2. Materials and Methods

### 2.1. Sample Isolation, Whole-Genome Sequencing, and Analysis of Genomic Features

Samples were isolated from tertiary wastewater effluent, collected from a wastewater treatment plant that uses (5 mg/L) chlorine for treatment in the North West Province, South Africa, as part of our laboratory routine ESKAPE pathogens monitoring program. An API 20E micro-organism identification kit (bioMerieux, Midrand, South Africa), and whole-genome sequencing were used to ascertain the identity of the isolates. Bacterial DNA was extracted from overnight broth cultures using a Nucleospin^®^ Tissue extraction kit (Macherey-Nagel, Düren, Germany) following the manufacturer’s protocol. Agarose gel electrophoresis and NanoDrop spectrophotometry (ND-100, NanoDrop Technologies Inc, Wilmington, DE, USA) were used to determine the integrity and the purity of the resultant DNA, respectively. Paired-end libraries were prepared from 1 ng bacterial DNA using a Nextera XT DNA Sample Preparation Kit and a Nextera XT Index kit (Illumina Inc., San Diego, California, USA) following the manufacturer’s protocol. Sequencing was performed using a MiSeq 2000 Illumina platform (250 bp paired-end reads). Raw sequence reads were quality filtered using FastQC; trimming was performed using Trimmomatic [13]. De novo assembling of the quality reads was performed using SPAdes version 3.13.0 [14]. CheckM [15] was used to assess the quality of the SPAdes assembled genomes. SPAdes assembled genomes were annotated by RAST [16] with default settings. 

### 2.2. Whole Genome-Based Taxonomic Analysis

For whole-genome-based taxonomic analysis, the SPAdes-assembled genomes were uploaded to the Type Strain Genome Server (TYGS) [17]. Pairwise comparison of the user-uploaded genomes (isolates S4 and S5) and the phylogenetically related type strains were performed using Genome Blast Distance Phylogeny (GBDP). Inter-genomic distances were inferred using the trimming algorithm and distance formula *d_5_*, with 100 replicates [18]. Digital DNA to DNA hybridization (dDDH) values and confidence intervals were calculated using the recommended settings of the Genome-to-Genome Distance Calculator (GGDC) 2.1. The resulting inter-genomic distances were used to infer a balanced minimum evolution tree with branch support via FASTME 2.1.4, including SPR post-processing [19]. The trees were rooted at the midpoint and visualized with PhyD3 [20]. FastANI version 0.1.2 [21] was used to estimate the average nucleotide identity (ANI) of the isolates using closely related genomes of *Enterobacter* species as reference genomes. 

### 2.3. Comparative Genomics

RAST annotated whole-genome sequences of species closely related to the isolates (S4 and S5) were uploaded onto the Kbase server for downstream analysis [22]. The used species were *E. asburiae* (CP011863), *Enterobacter bugandensis* (GCA_015137655), *Enterobacter cancerogenus* (GCA_000478345), *Enterobacter chengduensis* (CP043318), *Enterobacter chuandaensis* (GCA_003594915), *E. cloacae* subsp. *dissolvens* (NC_018079), *E. cloacae* subsp. *cloacae* (CP001918), *E. hormaechei* subsp. *hormaechei* (GCA_001875655), *E. hormaechei* subsp. *steigerwaltii* (CP017179), *E. hormaechei* subsp. *hoffmannii* (CP017186), *Enterobacter huaxiensis* (RWHU01000000), *E. kobei* (CP017181), *E. ludwigii* (CP017279.1), *Enterobacter mori* (GCA_000211415), *Enterobacter oligotrophica* (AP019007), *Enterobacter quasihormaechei* (GCA_004331385), *Enterobacter roggenkampii* (CP017184), *Enterobacter sichuanensis* (GCA_002939185), *Enterobacter soli* (GCA_001654845)*, Enterobacter timonensis* (GCA_900021175), *Enterobacter wuhouensis* (GCA_004331265) and *Enterobacter xiangfangensis* (CP017183) Domains in the genome sets were annotated using HMMER version 3.1 b [23], Gapped BLAST and PSI-BLAST [24]. The annotated domains were used to view the difference in functional roles among the genomes based on The SEED and COG (https://www.ncbi.nlm.nih.gov/research/cog-project/, accessed on 25 January 2021) categories/roles.

## 3. Results and Discussions

### 3.1. Genomic Features and Assembling Metrics of the Genomes of the Isolates

The genomes assembled with SPAdes resulted in high-quality genomes, as shown in Table 1. CheckM quality assessment of the assembled genomes showed that the genomes of isolates S4 and S5 had an estimated 97.43% and 94.67% completeness, respectively, and had 0.08% predicted contamination. Isolate S4 had a genome size of 4,658,088 bp, which corresponds to an N50 of 89,616 bp, having 71 contigs. Isolate S5 had a genome size of 4,442,534 bp, which corresponds to an N50 of 39,615 bp, having 132 contigs. The GC content was homogeneous with S4 and S5 having a GC content of 55.26% and 55.35%, respectively. RAST annotation of the genomes predicted that isolate S4 had 4545 coding genes while isolate S5 had 4341 coding genes. The genes for S4 were predicted to have 3997 distinct functions, with 1991 of the genes having SEED annotation ontology across 1806 distinct SEED functions. For S5, there were 3651 predicted distinct functions, with 1930 of the genes having SEED annotation ontology across 1751 distinct SEED functions.

### 3.2. Whole-Genome-Based Taxonomic Analysis

**Figure 1:**
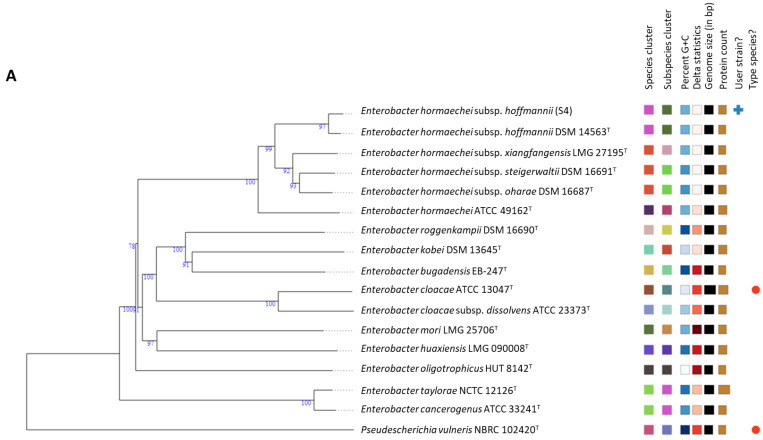

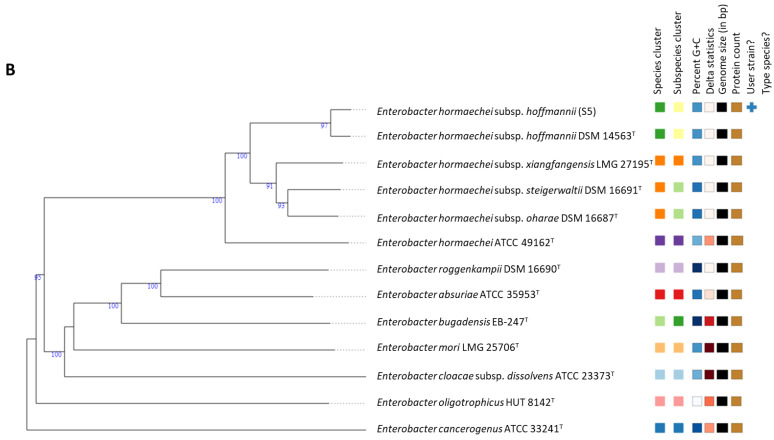
Trees inferred with FastME 2.1.6.1 from the Genome Blast Distance Phylogeny (GBDP) distances calculated from whole-genome sequences. Isolates S4 (**A**) and S5 (**B**) form a species cluster with *Enterobacter hormaechei* subsp. *hoffmannii,* having a dDDH of >70%, a clear indication that the isolates belong to the same species as *E. hormaechei* subsp. *hoffmannii*.

Taxonomic classification in the genus *Enterobacter* has been problematic, since some species and most subspecies have been assigned based on low-resolution methods [8,9]. In addition, this complexity has been made worse by the fact that phenotype-based methods cause misidentification of *Enterobacter* species and are unreliable for precise species identification [6]. To correctly classify our isolates (S4 and S5), we applied a more robust whole-genome-based taxonomic classification through comparison of the whole-genome sequences of our isolates with the whole-genome sequences of established type strains; for this purpose, we used the Type Strain Genome Server (TYGS) [17]. The TYGS results showed that isolates S4 and S5 form a highly supported clade corresponding to a species cluster with *Enterobacter hormaechei* subsp. *hoffmannii* (Figure 1). The main criterion for species affiliation is the 70% DNA–DNA hybridization threshold [25,26]. Digital DNA-DNA hybridization (dDDH) computed by the Genome–Genome Distance Calculator (GGDC) between the whole genomes of our isolates (S4 and S5) and that of *E. hormaechei* subsp. *hoffmannii* was well above the species cut-off point of 70%: 92.8% and 92.9%, respectively (Table 2), indicating that isolates S4 and S5 are indeed *E. hormaechei* subsp. *hoffmannii*. The GGDC results showed low delta values coupled with high average branch support, which denotes a high phylogenetic accuracy [27,28]. Another overall genome relatedness index important for the delineation of species based upon genome similarity is average nucleotide identity (ANI). This is one of the most important indices, since sequence identity has clockwise-like properties and provides the likelihood of correlation with times of divergence [29]. ANI is widely acknowledged as a measure of genomic relatedness, with an ANI ≥ 96% being the proposed cut-off point for species delineation [30]. In the present study, the ANI of the isolates S4 and S5 were well above the cut-off point compared to the closely phylogenetically related species *E. hormaechei* subsp. *hoffmannii* (Supplementary Table S1), indicating that the isolates used in this study are *E. hormaechei* subsp. *hoffmannii*. 

Based on ANI and dDDH, the taxonomic classification analysis from this study supports the recent report that *E. cloacae* subsp. *dissolvens* and *E. hormaechei* subsp. *hoffmannii* are species rather than subspecies; and *E. xiangfangensis*, *E. hormaechei* subsp. *oharae*, and *E. hormaechei* subsp. *steigerwaltii* are the same species (Supplementary Table S1). Based on the recently updated taxonomic classification of the genus *Enterobacter* by Wu et al. (2020), isolates S4 and S5 are *E. hoffmannii* [7]. Whole-genome sequences have greatly improved the identification of species as they elucidate functional profiles of taxonomic groups, and easily resolve ambiguities in the phylogeny of higher taxa, which would have been difficult through traditional approaches [11,12]. Low-resolution methods were applied in the earlier classification of *Enterobacter* species [8,9,10], which caused the above-mentioned inconsistency; more robust methods should be applied when classifying *Enterobacter* species.

### 3.3. Predictions of Potential Human Pathogenicity, Resistome, Virulome, and Biosynthetic Gene Clusters

Identifying pathogenic bacterial strains and understanding the biological mechanisms of pathogenicity is important for timely intervention programs, designing control strategies, as well as the development of targeted vaccines. To predict the pathogenicity of our isolates, we used the PathogenFinder algorithm [31]; both S4 and S5 were predicted to be human pathogens with a probability of greater than 0.78. The genome sequence of isolate S4 matched a total of 63 pathogenic families from a broad range of pathogens, including *Yersinia pestis*, *Salmonella enterica* subsp. *arizonae*, several pathogenic *Escherichia coli* strains, *Klebsiella pneumonia* strains, *Citrobacter koseri*, *Shigella boydii*, and *Enterobacter sakazakii*. Supplementary Materials Supporting Information 1a provides a detailed description of the matched families, which includes location (chromosomal or plasmid), completeness, and identity percentage for S4. Isolate S5 matched a total of 58 pathogenic families; Supplementary Materials Supporting Information 1b gives a detailed description of the matched families.

**Figure 2:**
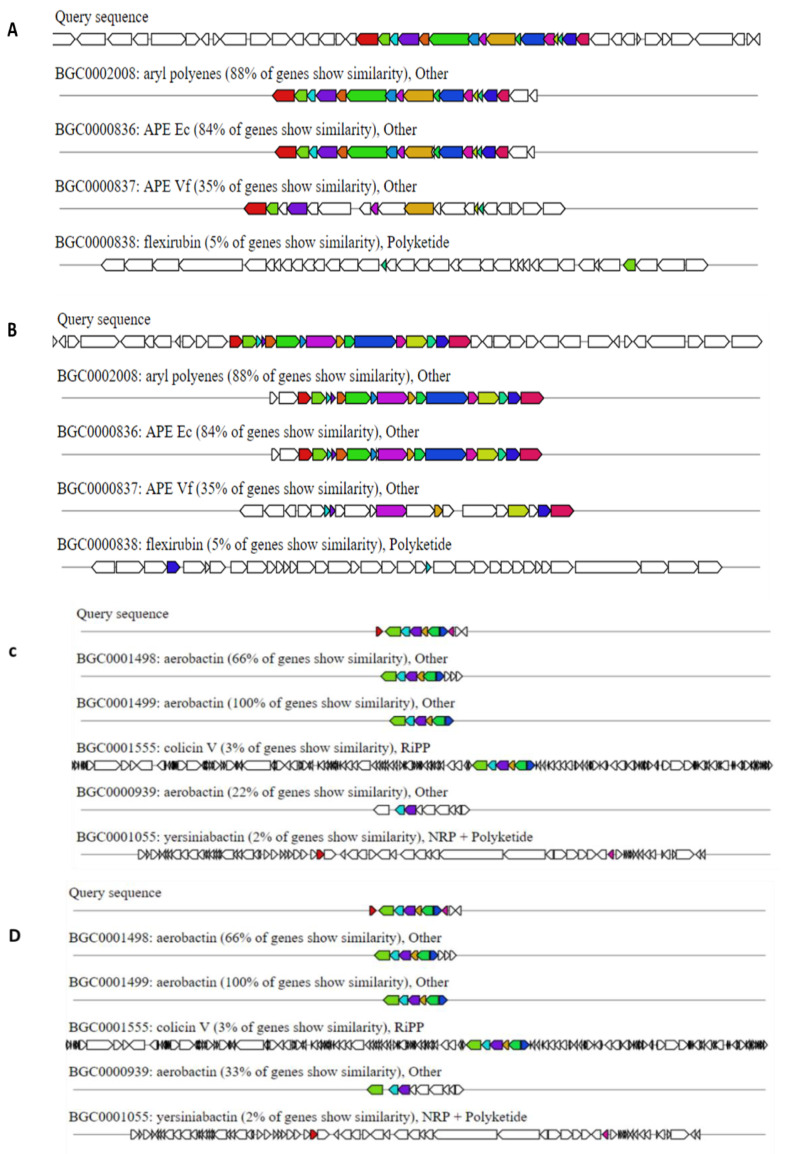
Biosynthetic gene clusters (BGCs) detected in the genome sequences of isolates S4 and S5. (**A**,**B**) Aryl polynes (APEs) for genomes S4 and S5, respectively. (**C**,**D**) Aerobactin for genomes S4 and S5, respectively. Query sequences are the genome sequences of the isolates S4 and S5.

For effective colonization of the host, pathogens require an arsenal of strategies to enable adherence, persistence, aggression, and evasion of innate and adaptive immunity [32,33,34]. We used the Virulence Factors of Pathogenic bacteria database to determine the presence of genes encoding the above-described arsenal. Both isolates S4 and S5 have the necessary virulence factors for successful host colonization and perpetuation of the pathogenesis process, supporting their potential pathogenicity (Table 3). In addition to the virulence factors, the isolates have several antibiotic resistance genes, which included genes encoding for beta-lactamase resistance. The mechanisms for antibiotic resistance were predicted to be antibiotics inactivation, antibiotic target alteration, reduced permeability to antibiotic, and antibiotic efflux (Supplementary Table S2). The presence of all the arsenal required for pathogenesis and the ability of the isolates to survive the chlorination process emphasize the importance of continuously monitoring wastewaters and evaluating the wastewater treatment process. A number of biosynthetic gene clusters have been shown to offer competitive fitness advantages and aid virulence. For example, aryl polyenes (APEs) were shown to function as fitness factors that increase protection from oxidative stress and contribute to biofilm formation [35]. In *E. coli*, APEs are present in most pathogenic strains, whereas they are typically absent from commensals and laboratory strains [36]. We used antiSMASH to identify the secondary metabolite biosynthetic gene clusters in our genome sequences [37]. Both S4 and S5 consist of aryl polyenes (Figure 2A,B); this could possibly explain their ability to survive the chlorination treatment process of the wastewater. We also identified aerobactin (Figure 2C,D), augmenting the predicted human pathogenic nature of the isolates. Lack of available iron is one of the first lines of defense to avoid bacterial infection [38,39,40]. Aerobactin, a hydroxymate-type siderophore, has been shown to offer a selective advantage under iron starvation and increases the virulence of *E. coli* strains [41,42].

### 3.4. Comparative Genomics

The development of next-generation sequencing technologies (NGS) has allowed for comparative genomics of multiple genomes. Comparative genomics, in particular pan-genome analysis, more accurately reflects the notion of bacterial species [11,43]. Pan-genome refers to the whole gene repertoire of a study group [44,45]. The pan-genome is divided into three components: (1) the core genome, which is a set of all genes common to all strains of the study; (2) non-core/accessory genome, which is a set of genes present in more than one, but not in all of the strains used in a study; and (3) singletons, which are genes unique to individual strains used in the study.

**Figure 3:**
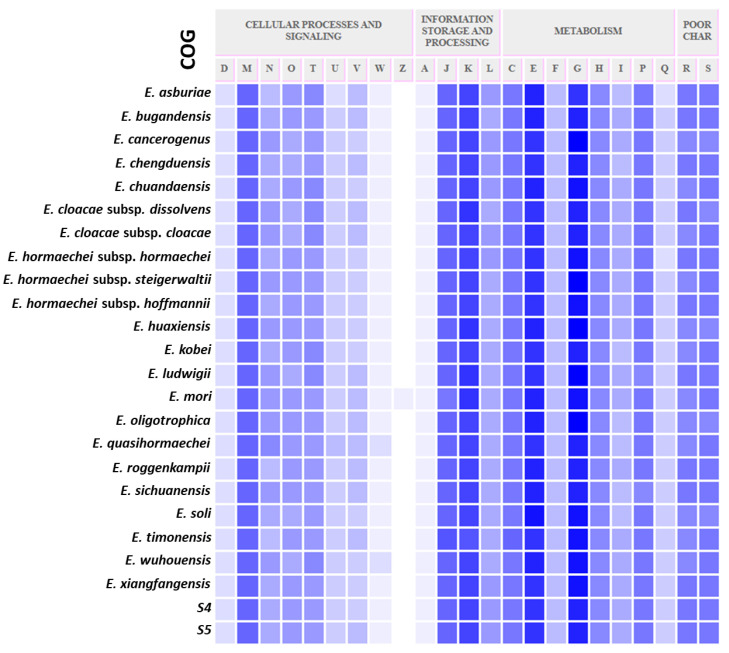
A heatmap showing clusters of orthologous genes (COGs) for isolates S4, S5, and a representative of all validly published *Enterobacter* species (see Materials and Methods for full species list and accession numbers). The COGs covered cellular processing and signaling (D: cell cycle control, cell division, chromosome partitioning; M: cell wall/membrane/envelope biogenesis; N: cell motility; O: post-translational modification, protein turnover, and chaperones; T: signal transduction mechanism; U: intracellular trafficking, secretion, and vesicular transportation; V: defense mechanisms; W: extracellular structures; Z: cytoskeleton), information storage and processing (A: RNA processing and modification; J: translation, ribosomal structure, and biogenesis; K: transportation; L: replication, recombination, and repair), metabolism (C: energy production and conversion; E: amino acid transport and metabolism; F: nucleotide transport and metabolism; G: carbohydrate transport and metabolism; H: coenzyme transport and metabolism; I: lipid transport and metabolism; P: inorganic ion transport and metabolism; Q: secondary metabolites biosynthesis, transport, and catabolism), as well as the poorly characterized (R and S).

**Figure 4:**
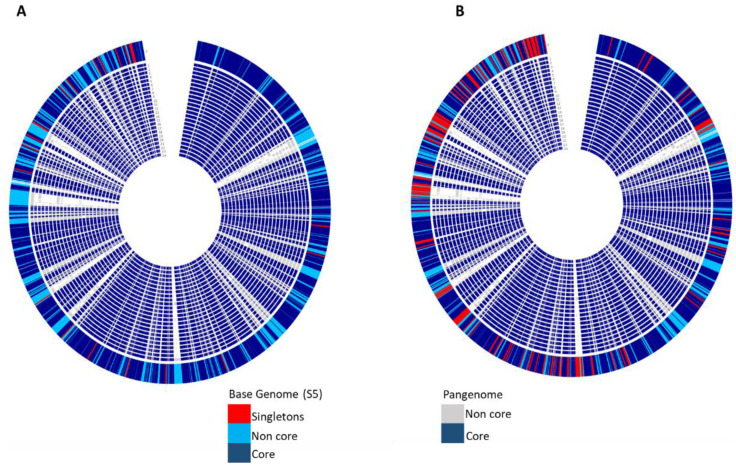
Pan-genome circular visualization output. (**A**) Isolates S4 and S5 compared to representatives of all validly published *Enterobacter* species (see Materials and Methods for full species list and accession numbers); each red strip indicates a gene that is unique to the isolate S5, which was used as a base genome. (**B**) Isolate S5 compared to validly published *Enterobacter* species; each red strip indicates a gene that is unique to the isolate S5. (**B**) Isolate S4 is not included in the analysis as it is highly identical to isolate S5 and would mask the true variation in the isolates compared to other *Enterobacter* species.

From the 24 *Enterobacter* genomes used in this study, there were a total of 6032 functions detected, of which 40.4% (2438) were core functions. The remaining 59.6% were part of the accessory genome. The core function genes covered the essential basics for survival; the clusters of orthologous genes (COGs) for the core functions are shown in Figure 3. They are homogeneously distributed, covering cellular processes and signaling, metabolism, and information storage and processing. Isolates S4 and S5 are more identical; thus, including all isolates in the pan-genome analysis masked how distinct the isolates are from other species (Figure 4A). However, removing one of the isolates (S4) revealed that the isolates are greatly distinct from other samples used in this study (Figure 4B), having a total of 234 singletons. Supplementary Materials Supporting Information 2 provides details of all the singletons for isolate S5 compared to all the other samples, excluding isolate S4. A large fraction of the accessory genomes is mobile genetic elements [45]. These accessory genomes place a host cell in an advantageous position to be viable under specific conditions [46,47]. The singletons comprise many virulence factors, antibiotic resistance genes, phage related proteins, with hypothetical proteins and genes of unknown function constituting 54% of the singletons. Of the singletons, some of the genes to note are (phage-encoded virulence determinant Bor, several phage related proteins/genes, type IV secretion complex proteins (VirB5, VirB6, VirB9, VirB1, VirD4, VirB2, VirB8, VirB10, VirB11), T6SS secretion lipoprotein TssJ (VasD), T1SS secreted agglutinin RTX, nitrilotriacetate monooxygenase component A (EC 1.14.13.-), multidrug resistance protein MdtH, SOS-response repressor and protease LexA (EC 3.4.21.88), inner-membrane proton/drug antiporter (MSF type) of tripartite multidrug efflux system, arsenite/antimonite pump-driving ATPase ArsA (EC 3.6.3.16), ferric hydroxamate outer membrane receptor FhuA, diguanylate cyclase/phosphodiesterase (GGDEF and EAL domains) with PAS/PAC sensor(s), and COG3121: P pilus assembly protein, chaperone PapD), a clear indication of the virulence nature of the isolates (Supplementary Materials Supporting Information 2). Expression of *bor* significantly increases the survival of the *Escherichia coli* host cell in animal serum. This property is a well-known bacterial virulence determinant [48,49]. Prophages are known to increase the virulence of pathogenic strains [50,51], and there is a positive correlation between the phage-related DNA content of a given Enterobacterium and its pathogenicity [52]. In this study, the singletons for S5 constitute a higher proportion of phage DNA, and the isolates were predicted to be human pathogens. Type IV secretion systems mediate the translocation of virulence factors (proteins and/or DNA) from Gram-negative bacteria into eukaryotic cells [53,54].

**Figure 5:**
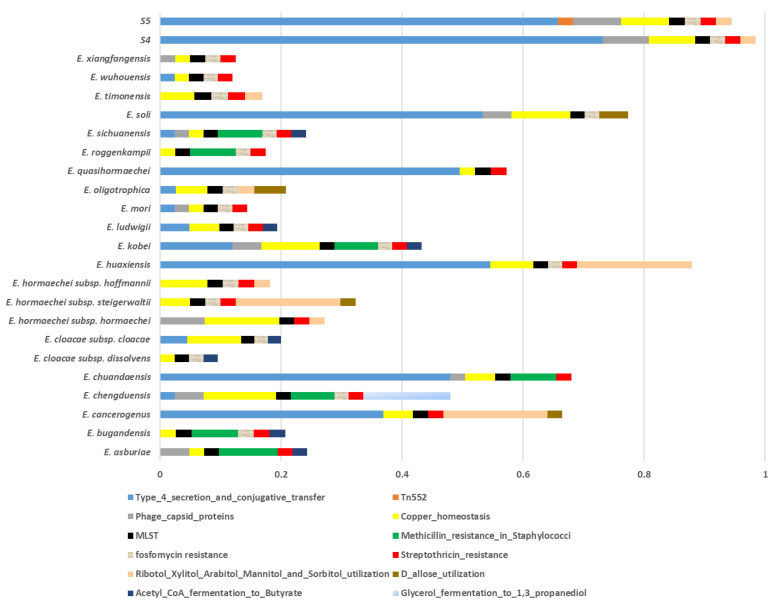
Distribution of a selected SEED domain categories between the isolates (S4, S5) and the representatives of all validly published *Enterobacter* species (see Materials and Methods for full species list and accession numbers). Interestingly, isolates S4 and S5 have the largest proportion of Type 4 Secretion System in addition to being the only genomes carrying Tn522.

Comparison of protein domain content allows for the construction of bacteria phylogeny independent of gene sequence [55], which is a better indicator of shared physiology and ecology [56]. This also takes into account the impacts of mutations, gene loss, and horizontal gene transfer. The domain architecture is preserved at large phylogenetic distances [57,58]. Protein domains are distinct, functional units responsible for specific functions and interactions; thus, we also applied the SEED protein domain-class content to study the species diversity. Under the SEED category virulence, subcategory Type_4_secretion_and_conjugative_transfer, isolates S4 and S5 have a higher proportion of domains related to this function (Figure 5). These are also relatively higher in *E. soli*, *E. quasihormoachei*, *E. huaxiansis*, *E. chuandensis*, and *E. cancerogenus* compared to other genomes, albeit lower than in the isolates S4 and S5. Moreover, S4 and S5 have a considerably higher level of phage_capsid_protein, with S5 being the only genome harboring Tn552. In addition, S5 shows a relatively higher level of domains related to copper homeostasis (Figure 5).

## 4. Conclusions

This study provides comprehensive insights into the genomic structure of *E. hormaechi* subsp. *hoffmmanii* from South Africa, and aids with the baseline of global surveillance and monitoring of *Enterobacter* species, a member of the clinically significant ESKAPE pathogens. From the genome sequences, several important features including virulence factors, antibiotic resistance genes, and biosynthetic gene clusters (BGCs) were explored. The genome sequences provide an important resource for future comparative genomics. Moreover, the study demonstrates the risks associated with wastewater and the need for continuous monitoring of the treated wastewaters to ensure efficacy in the removal of pathogens. It also helps local authorities to understand the potential dangers associated with the wastewater, and encourage them to reconsider their treatment processes. The results from whole-genome taxonomic classification support recent updates in the taxonomy of the genus *Enterobacter*, emphasizing the importance of using high-resolution analytic methods for species identification.

## Figures and Tables

**Table 1:** Quality metrics of the isolates assembled genomes.

Isolate	S4	S5
Total Contigs	71	132
Total Length (bp)	4,658,088	4,442,534
Largest Contig (bp)	329,202	135,452
N50 (bp)	89,616	39,615
GC%	55.26	55.35
Coding Genes	4545	4341
Non Coding Repeats	16	10
Non Coding RNA	16	56

**Table 2:** Type Strain Genome Server calculation of digital DNA–DNA hybridizations of isolates (S4, S5), and some of their closely related type strains.

Query Strain	Subject Strain	dDDH (d4, in %)	C.I. (d4, in %)	G + C Content Difference (in %)
S4SPAdes.contigs.fa	*Enterobacter hormaechei* subsp. *hoffmannii* DSM 14563^T^	92.9	[90.9–94.4]	0.07
S5SPAdes.contigs.fa	*Enterobacter hormaechei* subsp. *hoffmannii* DSM 14563^T^	92.8	[90.8–94.4]	0.01
S4SPAdes.contigs.fa	*Enterobacter hormaechei* subsp. *oharae* DSM 16687^T^	66.	[63.4–69.2]	0.32
S5SPAdes.contigs.fa	*Enterobacter hormaechei* subsp. *oharae* DSM 16687^T^	66.4	[63.5–69.3]	0.24
S4SPAdes.contigs.fa	*Enterobacter hormaechei* subsp. *xiangfangensis* LMG 27195^T^	66.1	[63.2–69.0]	0.02
S5SPAdes.contigs.fa	*Enterobacter hormaechei* subsp. *xiangfangensis* LMG 27195^T^	66.2	[63.2–69.0]	0.07
S4SPAdes.contigs.fa	*Enterobacter hormaechei* subsp. *steigerwaltii* DSM 16691^T^	66	[63.0–68.8]	0.29
S5SPAdes.contigs.fa	*Enterobacter hormaechei* subsp. *steigerwaltii* DSM 16691^T^	65.9	[62.9–68.7]	0.21
S4SPAdes.contigs.fa	*Enterobacter hormaechei* ATCC 49162^T^	57.3	[54.5–60.0]	0.02
S5SPAdes.contigs.fa	*Enterobacter hormaechei* ATCC 49162^T^	57.3	[54.5–60.1]	0.1
S4SPAdes.contigs.fa	*Enterobacter bugandensis* EB-247^T^	35.1	[32.7–37.6]	0.74
S5SPAdes.contigs.fa	*Enterobacter bugandensis* EB-247^T^	35.3	[32.8–37.8]	0.65
S4SPAdes.contigs.fa	*Enterobacter mori* LMG 25706^T^	34.2	[31.7–36.7]	0.04
S5SPAdes.contigs.fa	*Enterobacter mori* LMG 25706^T^	34.3	[31.9–36.8]	0.05
S4SPAdes.contigs.fa	*Enterobacter roggenkampii* DSM 16690^T^	33.7	[31.3–36.3]	0.78
S5SPAdes.contigs.fa	*Enterobacter roggenkampii* DSM 16690^T^	34	[31.5–36.5]	0.7
S4SPAdes.contigs.fa	*Enterobacter oligotrophicus* HUT 8142^T^	33.4	[31.0–35.9]	0.96
S5SPAdes.contigs.fa	*Enterobacter oligotrophicus* HUT 8142^T^	33.5	[31.1–36.0]	1.04
S4SPAdes.contigs.fa	*Enterobacter cloacae* subsp. *dissolvens* ATCC 23373^T^	32	[29.6–34.5]	0.1
S5SPAdes.contigs.fa	*Enterobacter cloacae* subsp. *dissolvens* ATCC 23373^T^	32.1	[29.7–34.6]	0.18
S4SPAdes.contigs.fa	*Enterobacter cancerogenus* ATCC 33241^T^	31.3	[28.9–33.8]	0.42
S5SPAdes.contigs.fa	*Enterobacter cancerogenus* ATCC 33241^T^	31.4	[29.0–34.0]	0.34

**Table 3:** Virulence factors detected in the genome sequences of isolates S4 and S5.

Virulence Factor Class	Virulence Factors	S4	S5
	Type 3 fimbriae	1	1
	Type I fimbriae	8	6
Adherence	Curli fibers(*Escherichia*)	3	2
	Hemorrhagic *E. coli* pilus (HCP) (*Escherichia*)	2	2
	Type IV (pili) (*Yersinia*)	1	1
Antiphagocytosis	Capsule	15	15
Efflux pump	AcrAB	3	3
	Aerobactin	5	5
	Ent Siderophore	12	12
	Salmochelin	1	1
Iron uptake	Heme transport (*Shigella*)	1	1
	Heme uptake (*Escherichia*)	2	2
	Iron/Manganese transport (*Escherichia*)	1	1
Regulation	RcsAB	2	1
	T6SS-I	10	9
	T6SS-II	4	4
	T6SS-III	0	2
	EPS type secretion system (*Vibrio*)	1	1
	Flagella (cluster I) (*Yersinia*)	32	32
Secretion system	Hcp secretion island-1 encoded type VI secretion system (H-T6SS) (*Pseudomonas*)	3	3
	SCI-I T6SS (*Escherichia*)	1	0
	T2SS (Yst1) (*Yersinia*)	1	1
	T2SS (*Aeromonas)*	1	1
	TTSS (SPI-encode) (*Salmonela*)	1	0
Serum resistance	LPS rfb locus	1	1
Toxin	Heat-stable cytotonic toxin	1	1
Autotransporter	EhaB	1	
Endotoxin	LOS (*Haemophilus)*	1	1
	Agf/Csg (*Salmonella*)	4	4
	Fim (*Salmonella*)	5	5
Fimbrial adherence determinants	Sti (*Salmonella*)	1	1
	Stj (*Salmonella*)	1	0
	Stk (*Salmonella*)	4	4
Invasion	Flagella (*Burkholderia*)	6	6
Motility	Flagella (*Bordetella*)	1	1

## Data Availability

The raw sequences generated for this study were deposited into the National Centre for Biotechnology Information (NCBI) database under the BioProject ID: PRJNA735620, with BioSample Accessions SAMN19590289, SAMN19590290. All the other data supporting the findings of this study are available in this published article and its supplementary information files.

## Supplementary Materials

The supplementary materials are available online at https://www.mdpi.com/article/10.3390/microorganisms9091928/s1.
