# Supplementary material for: Whole Genome Sequencing Based Taxonomic Classification, and Comparative Genomic Analysis of Potentially Human Pathogenic Enterobacter spp. Isolated from Chlorinated Wastewater in the North West Province, South Africa
Source: Microorganisms. 2021 Sep 10;9(9):1928. doi: 10.3390/microorganisms9091928 (PMC8466087; doi:10.3390/microorganisms9091928)
Supplement: Supplementary file 1 [file microorganisms-09-01928-s001.zip › microorganisms-1364401-supplementary revised version.pdf]

Supporting Information 1a. Pathogenic Families Matched by isolate S4

Organism	Accession Number	Matched Pathogenic Protein Family	Identity %
Yersinia pestis Pestoides F plasmid MT, complete sequence.	CP000670	conjugative transfer oriT nicking-unwinding protein	98
Yersinia pestis biovar Microtus str. 91001 plasmid pMT1, complete sequence.	AE017045	putative DNA-binding protein	96.99
Salmonella enterica subsp. arizonae serovar 62:z4,z23:--, complete genome.	CP000880	hypothetical protein	97.07
Escherichia coli str. K12 substr. DH10B, complete genome.	CP000948	CP4-6 prophage; predicted dehydratase	97.86
Escherichia fergusonii ATCC 35469 chromosome, complete genome.	CU928158	inorganic polyphosphate/ATP-NAD kinase	96.23
Klebsiella pneumoniae NTUH-K2044 DNA, complete genome.	AP006725	4-hydroxyphenylacetate 3-hydroxylase	97.88
Escherichia coli IAI39 chromosome, complete genome.	CU928164	Group II intron-encoded reverse transcriptase/maturase	100
Citrobacter koseri ATCC BAA-895, complete genome.	CP000822	hypothetical protein	97.05
Escherichia coli UTI89, complete genome.	CP000243	galactose-proton symporter	95.27
Klebsiella pneumoniae 342, complete genome.	CP000964	ascorbate-specific permease IIC component	96.14
Escherichia coli str. K12 substr. DH10B, complete genome.	CP000948	CP4-6 prophage; predicted sugar transporter	98.7
Salmonella enterica subsp. arizonae serovar 62:z4,z23:--, complete genome.	CP000880	hypothetical protein	96.8
Yersinia pestis biovar Microtus str. 91001 plasmid pMT1, complete sequence.	AE017045	putative partitioning protein A	97.26
Klebsiella pneumoniae subsp. pneumoniae MGH 78578, complete sequence.	CP000647	putative permease (MFS superfamily)	95.88
Escherichia coli UTI89, complete genome.	CP000243	Nucleoside permease NupC	95.19
Citrobacter koseri ATCC BAA-895, complete genome.	CP000822	hypothetical protein	96.69
Klebsiella pneumoniae NTUH-K2044 DNA, complete genome.	AP006725	pyrroloquinoline quinone synthesis protein E	95.53
Salmonella enterica subsp. enterica serovar Dublin str. C	CP001143	2021853 plasmid pCT0202185	96.92
Escherichia coli 042 complete genome.	FN554766	putative transposase	99.72
Shigella boydii CDC 3083-94, complete genome.	CP001063	DNA replication and repair protein RecF	95.24
Escherichia coli APEC O1, complete genome.	CP000468	UDP-galactose-4-epimerase	95.27
Klebsiella pneumoniae subsp. pneumoniae MGH 78578, complete sequence.	CP000647	3-phenylpropionate dioxygenase, alpha subunit	95.59
Salmonella enterica subsp. arizonae serovar 62:z4,z23:--, complete genome.	CP000880	hypothetical protein	97.34
Citrobacter koseri ATCC BAA-895, complete genome.	CP000822	hypothetical protein	96.66
Citrobacter koseri ATCC BAA-895, complete genome.	CP000822	hypothetical protein	95.38
Escherichia coli UTI89, complete genome.	CP000243	30S ribosomal protein S2	97.51
Escherichia fergusonii ATCC 35469 chromosome, complete genome.	CU928158	RNA polymerase, sigma S (sigma 38) factor	99.31
Shigella flexneri 5 str. 8401, complete genome.	CP000266	conserved hypothetical protein	96.23
Klebsiella pneumoniae 342, complete genome.	CP000964	3,4-dihydroxyphenylacetate 2,3-dioxygenase	95.41
Salmonella enterica subsp. enterica serovar Choleraesuis str. SC-B67, complete genome.	AE017220	transcriptional regulation of aerobic, anaerobic respiration, osmotic balance (CRP family)	98
Citrobacter koseri ATCC BAA-895, complete genome.	CP000822	hypothetical protein	98.84
Yersinia pestis Pestoides F plasmid MT, complete sequence.	CP000670	conserved hypothetical protein	99.24
Citrobacter koseri ATCC BAA-895, complete genome.	CP000822	hypothetical protein	98.46
Yersinia pestis biovar Microtus str. 91001 plasmid pMT1, complete sequence.	AE017045	hypothetical protein	97.3
Salmonella enterica subsp. enterica serovar Paratyphi C strain RKS4594, complete genome.	CP000857	L-ribulose-5-phosphate 4-epimerase	95.67
Yersinia pestis Pestoides F plasmid MT, complete sequence.	CP000670	conjugative transfer fimbrial acetylation protein	99.59

Continuation Supporting Information 1a

Salmonella enterica subsp. enterica serovar Newport str. SL254, complete genome.	CP001113	ribonuclease III	95.82
Salmonella enterica subsp. arizonae serovar 62:z4,z23:--, complete genome.	CP000880	hypothetical protein	98.55
Escherichia coli 536, complete genome.	CP000247	oligoribonuclease	95.58
Salmonella enterica subsp. enterica serovar Choleraesuis str. SC-B67, complete genome.	AE017220	putative hexulose phosphate synthase (arabino hexulose phosphate formaldehyde lyase)	96.3
Yersinia pestis biovar Microtus str. 91001 plasmid pMT1, complete sequence.	AE017045	putative DNA methylase	100
Enterobacter sakazakii ATCC BAA-894, complete genome.	CP000783	hypothetical protein	98.27
Salmonella enterica subsp. enterica serovar Schwarzengrund str. CVM19633, complete genome.	CP001127	flavodoxin	96.02
Escherichia coli UTI89, complete genome.	CP000243	RNA polymerase sigma E	98.95
Citrobacter koseri ATCC BAA-895, complete genome.	CP000822	hypothetical protein	97.35
Salmonella enterica subsp. enterica serovar Dublin str. C	CP001144	2021853, complete genome.	97.86
Yersinia pestis biovar Microtus str. 91001 plasmid pMT1, complete sequence.	AE017045	putative antirestriction protein	95.24
Escherichia coli UTI89, complete genome.	CP000243	50S ribosomal subunit protein L13	97.89
Yersinia pestis biovar Microtus str. 91001 plasmid pMT1, complete sequence.	AE017045	hypothetical protein	98.56
Enterobacter sakazakii ATCC BAA-894, complete genome.	CP000783	hypothetical protein	96.88
Escherichia coli SMS-3-5, complete genome.	CP000970	putative inner membrane protein YqjF	95.38
Klebsiella pneumoniae NTUH-K2044 DNA, complete genome.	AP006725	transcriptional repressor for methionine biosynthesis	97.14
Salmonella enterica subsp. enterica serovar Paratyphi C strain RKS4594, complete genome.	CP000857	co-chaperonin GroES	96.91
Salmonella enterica subsp. enterica serovar Typhi str. CT18	XXX	XXX	100
Escherichia coli 536, complete genome.	CP000247	thioredoxin 1	98.17
Escherichia coli 042 complete genome.	FN554766	ribosome-binding factor A	95.52
Escherichia coli str. K12 substr. DH10B, complete genome.	CP000948	CP4-6 prophage; partial transposase of insertion element IS911A	100
Enterobacter sakazakii ATCC BAA-894 plasmid pESA2, complete sequence.	CP000784	hypothetical protein	100
Salmonella enterica subsp. enterica serovar Choleraesuis str. SC-B67, complete genome.	AE017220	chemotaxis regulator, transmits chemoreceptor signals to flagelllar motor components	95.35
Klebsiella pneumoniae NTUH-K2044 DNA, complete genome.	AP006725	preprotein translocase auxillary subunit	99.09
Enterobacter sakazakii ATCC BAA-894 plasmid pESA2, complete sequence.	CP000784	hypothetical protein	100
Salmonella enterica subsp. arizonae serovar 62:z4,z23:--, complete genome.	CP000880	hypothetical protein	97.44
Salmonella enterica subsp. enterica serovar Newport str. SL254, complete genome.	CP001113	ascorbate-specific phosphotransferase enzyme IIB component	98.02
Salmonella enterica subsp. arizonae serovar 62:z4,z23:--, complete genome.	CP000880	hypothetical protein	97.98
Escherichia coli UTI89, complete genome.	CP000243	conserved hypothetical protein YihD	95.51
Klebsiella pneumoniae NTUH-K2044 DNA, complete genome.	AP006725	cold shock protein	98.55
Salmonella enterica subsp. enterica serovar Paratyphi A str. ATCC 9150, complete [illegible]	[illegible]	[illegible]	[illegible]

Supporting Information 1b. Pathogenic Families Matched by Isolate S5

Organism	Accession Number	Matched Pathogenic Protein Family	Identity %
Yersinia pestis biovar Microtus str. 91001 plasmid pMT1, complete sequence.	AE017045	putative DNA-binding protein	96.99
Salmonella enterica subsp. arizonae serovar 62:z4,z23:--, complete genome.	CP000880	hypothetical protein	97.07
Escherichia coli str. K12 substr. DH10B, complete genome.	CP000948	CP4-6 prophage; predicted dehydratase	97.86
Escherichia fergusonii ATCC 35469 chromosome, complete genome.	CU928158	inorganic polyphosphate/ATP-NAD kinase	96.23
Klebsiella pneumoniae NTUH-K2044 DNA, complete genome.	AP006725	4-hydroxyphenylacetate 3-hydroxylase	97.88
Citrobacter koseri ATCC BAA-895, complete genome.	CP000822	hypothetical protein	97.05
Escherichia coli UTI89, complete genome.	CP000243	galactose-proton symporter	95.27
Klebsiella pneumoniae 342, complete genome.	CP000964	ascorbate-specific permease IIC component	96.14
Escherichia coli str. K12 substr. DH10B, complete genome.	CP000948	CP4-6 prophage; predicted sugar transporter	98.7
Salmonella enterica subsp. arizonae serovar 62:z4,z23:--, complete genome.	CP000880	hypothetical protein	96.8
Yersinia pestis biovar Microtus str. 91001 plasmid pMT1, complete sequence.	AE017045	putative partitioning protein A	97.26
Klebsiella pneumoniae subsp. pneumoniae MGH 78578, complete sequence.	CP000647	putative permease (MFS superfamily)	95.88
Escherichia coli UTI89, complete genome.	CP000243	Nucleoside permease NupC	95.19
Citrobacter koseri ATCC BAA-895, complete genome.	CP000822	hypothetical protein	96.69
Klebsiella pneumoniae NTUH-K2044 DNA, complete genome.	AP006725	pyrroloquinoline quinone synthesis protein E	95.53
Salmonella enterica subsp. enterica serovar Dublin str. C	CP001143	2021853 plasmid pCT0202185	96.92
Shigella boydii CDC 3083-94, complete genome.	CP001063	DNA replication and repair protein RecF	95.24
Escherichia coli APEC O1, complete genome.	CP000468	UDP-galactose-4-epimerase	95.27
Salmonella enterica subsp. arizonae serovar 62:z4,z23:--, complete genome.	CP000880	hypothetical protein	97.34
Citrobacter koseri ATCC BAA-895, complete genome.	CP000822	hypothetical protein	96.66
Citrobacter koseri ATCC BAA-895, complete genome.	CP000822	hypothetical protein	95.38
Escherichia coli UTI89, complete genome.	CP000243	30S ribosomal protein S2	97.51
Shigella flexneri 2002017, complete genome.	CP001383	ISEhe3, transposase orfB	95.19
Escherichia fergusonii ATCC 35469 chromosome, complete genome.	CU928158	RNA polymerase, sigma S (sigma 38) factor	96.57
Shigella flexneri 5 str. 8401, complete genome.	CP000266	conserved hypothetical protein	96.23
Klebsiella pneumoniae 342, complete genome.	CP000964	3,4-dihydroxyphenylacetate 2,3-dioxygenase	95.41
Escherichia coli E24377A, complete genome.	CP000800	pyruvate formate-lyase 1-activating enzyme	97.15
Citrobacter koseri ATCC BAA-895, complete genome.	CP000822	hypothetical protein	98.84
Citrobacter koseri ATCC BAA-895, complete genome.	CP000822	hypothetical protein	98.46
Yersinia pestis biovar Microtus str. 91001 plasmid pMT1, complete sequence.	AE017045	hypothetical protein	97.3
Salmonella enterica subsp. enterica serovar Paratyphi C strain RKS4594, complete genome.	CP000857	L-ribulose-5-phosphate 4-epimerase	95.67

Continuation Supporting Information 1b

Salmonella enterica subsp. enterica serovar Newport str. SL254, complete genome.	CP001113	ribonuclease III	96.9
Salmonella enterica subsp. arizonae serovar 62:z4,z23:-- , complete genome.	CP000880	hypothetical protein	98.55
Escherichia coli 536, complete genome.	CP000247	oligoribonuclease	95.58
Salmonella enterica subsp. enterica serovar Choleraesuis str. SC-B67, complete genome.	AE017220	putative hexulose phosphate synthase (arabino hexulose phosphate formaldehyde lyase)	96.3
Yersinia pestis biovar Microtus str. 91001 plasmid pMT1, complete sequence.	AE017045	putative DNA methylase	100
Klebsiella pneumoniae NTUH-K2044 plasmid pK2044 DNA, complete genome.	AP006726	hypothetical orf in IS2	96.03
Enterobacter sakazakii ATCC BAA-894, complete genome.	CP000783	hypothetical protein	98.27
Salmonella enterica subsp. enterica serovar Schwarzengrund str. CVM19633, complete genome.	CP001127	flavodoxin	96.02
Escherichia coli UTI89, complete genome.	CP000243	RNA polymerase sigma E	98.95
Shigella dysenteriae Sd197, complete genome.	CP000034	conserved hypothetical protein	95
Citrobacter koseri ATCC BAA-895, complete genome.	CP000822	hypothetical protein	97.35
Salmonella enterica subsp. enterica serovar Dublin str. C	CP001144	2021853, complete genome.	97.86
Salmonella enterica subsp. enterica serovar Paratyphi B str. SPB7, complete genome.	CP000886	hypothetical protein	95.04
Yersinia pestis biovar Microtus str. 91001 plasmid pMT1, complete sequence.	AE017045	putative antirestriction protein	95.24
Yersinia pestis biovar Microtus str. 91001 plasmid pMT1, complete sequence.	AE017045	hypothetical protein	98.56
Enterobacter sakazakii ATCC BAA-894, complete genome.	CP000783	hypothetical protein	96.88
Escherichia coli SMS-3-5, complete genome.	CP000970	putative inner membrane protein YqjF	95.38
Klebsiella pneumoniae NTUH-K2044 DNA, complete genome.	AP006725	transcriptional repressor for methionine biosynthesis	97.14
Salmonella enterica subsp. enterica serovar Paratyphi C strain RKS4594, complete genome.	CP000857	co-chaperonin GroES	96.91
Escherichia coli 536, complete genome.	CP000247	thioredoxin 1	98.17
Escherichia coli 042 complete genome.	FN554766	ribosome-binding factor A	95.52
Salmonella enterica subsp. enterica serovar Choleraesuis str. SC-B67, complete genome.	AE017220	chemotaxis regulator, transmits chemoreceptor signals to flagelllar motor components	95.35
Salmonella enterica subsp. enterica serovar Newport str. SL254, complete genome.	CP001113	histidine triad nucleotide-binding protein 2	94.96
Salmonella enterica subsp. enterica serovar Paratyphi A str. ATCC 9150, complete genome.	CP000026	cell division protein FtsL	95.04
Klebsiella pneumoniae NTUH-K2044 DNA, complete genome.	AP006725	preprotein translocase auxillary subunit	99.09
Salmonella enterica subsp. arizonae serovar 62:z4,z23:-- , complete genome.	CP000880	hypothetical protein	97.44
Salmonella enterica subsp. enterica serovar Newport str. SL254, complete genome.	CP001113	ascorbate-specific phosphotransferase enzyme IIB component	98.02
Salmonella enterica subsp. arizonae serovar 62:z4,z23:-- , complete genome.	CP000880	hypothetical protein	97.98
Escherichia coli UTI89, complete genome.	CP000243	conserved hypothetical protein YihD	95.51
Klebsiella pneumoniae NTUH-K2044 DNA, complete genome.	AP006725	cold shock protein	98.55
Salmonella enterica subsp. enterica serovar Paratyphi A str. ATCC 9150, complete genome.	CP000026	conserved hypothetical protein	98.53
Shigella dysenteriae Sd197, complete genome.	CP000034	conserved hypothetical protein	97.01
Yersinia pestis Pestoides F plasmid MT, complete sequence.	CP000670	conjugative transfer fimbrial synthesis protein	98.78
Klebsiella pneumoniae NTUH-K2044 DNA, complete genome.	AP006725	putative cytoplasmic protein	95.89
Citrobacter koseri ATCC BAA-895, complete genome.	CP000822	hypothetical protein	96.1
Klebsiella pneumoniae NTUH-K2044 DNA, complete genome.	AP006725	putative amino acid/amine transport protein	98.11
Yersinia pestis biovar Microtus str. 91001 plasmid pMT1, complete sequence.	AE017045	hypothetical protein	98.63
