# Supplementary material for: Whole Genome Sequencing Based Taxonomic Classification, and Comparative Genomic Analysis of Potentially Human Pathogenic Enterobacter spp. Isolated from Chlorinated Wastewater in the North West Province, South Africa
Source: Microorganisms. 2021 Sep 10;9(9):1928. doi: 10.3390/microorganisms9091928 (PMC8466087; doi:10.3390/microorganisms9091928)
Supplement: Supplementary file 1 [file microorganisms-09-01928-s001.zip › Supplementary Tables.pptx]

## Slide 1
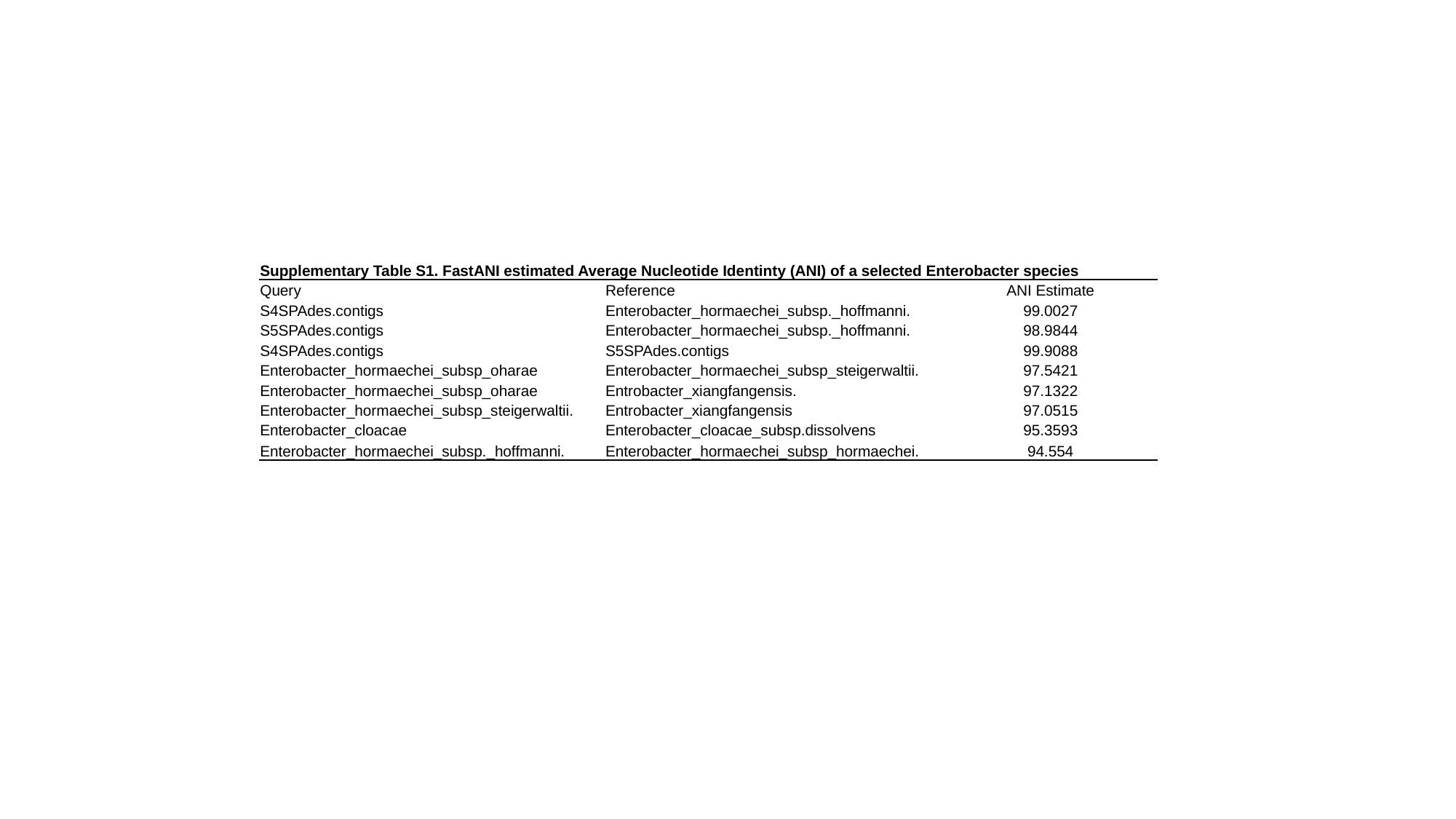

| Supplementary Table S1. FastANI estimated Average Nucleotide Identinty (ANI) of a selected Enterobacter species | | |
| --- | --- | --- |
| Query | Reference | ANI Estimate |
| S4SPAdes.contigs | Enterobacter\_hormaechei\_subsp.\_hoffmanni. | 99.0027 |
| S5SPAdes.contigs | Enterobacter\_hormaechei\_subsp.\_hoffmanni. | 98.9844 |
| S4SPAdes.contigs | S5SPAdes.contigs | 99.9088 |
| Enterobacter\_hormaechei\_subsp\_oharae | Enterobacter\_hormaechei\_subsp\_steigerwaltii. | 97.5421 |
| Enterobacter\_hormaechei\_subsp\_oharae | Entrobacter\_xiangfangensis. | 97.1322 |
| Enterobacter\_hormaechei\_subsp\_steigerwaltii. | Entrobacter\_xiangfangensis | 97.0515 |
| Enterobacter\_cloacae | Enterobacter\_cloacae\_subsp.dissolvens | 95.3593 |
| Enterobacter\_hormaechei\_subsp.\_hoffmanni. | Enterobacter\_hormaechei\_subsp\_hormaechei. | 94.554 |
| | | |

## Slide 2
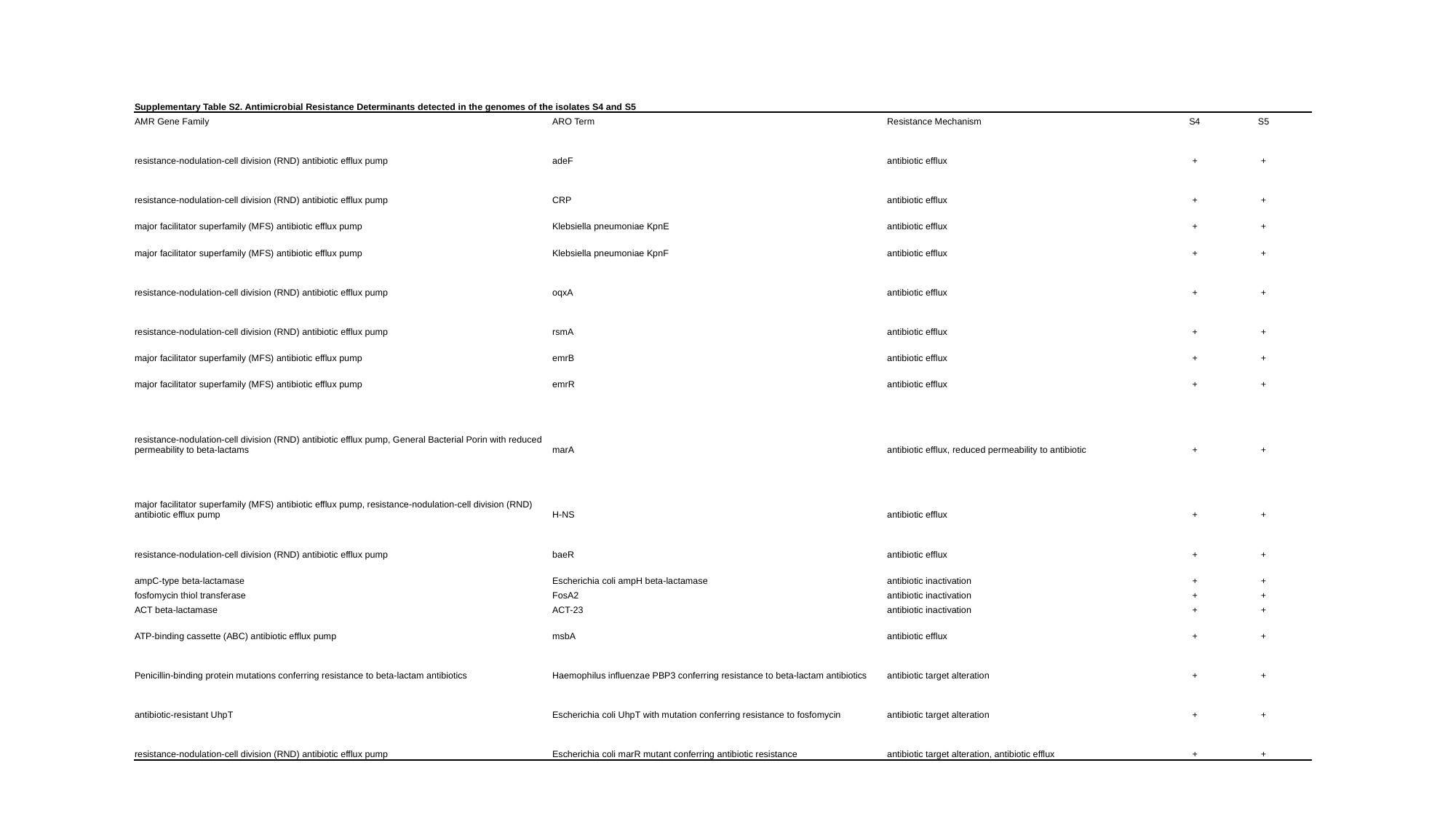

| Supplementary Table S2. Antimicrobial Resistance Determinants detected in the genomes of the isolates S4 and S5 | | | | | |
| --- | --- | --- | --- | --- | --- |
| AMR Gene Family | ARO Term | Resistance Mechanism | S4 | S5 | |
| resistance-nodulation-cell division (RND) antibiotic efflux pump | adeF | antibiotic efflux | + | + | |
| resistance-nodulation-cell division (RND) antibiotic efflux pump | CRP | antibiotic efflux | + | + | |
| major facilitator superfamily (MFS) antibiotic efflux pump | Klebsiella pneumoniae KpnE | antibiotic efflux | + | + | |
| major facilitator superfamily (MFS) antibiotic efflux pump | Klebsiella pneumoniae KpnF | antibiotic efflux | + | + | |
| resistance-nodulation-cell division (RND) antibiotic efflux pump | oqxA | antibiotic efflux | + | + | |
| resistance-nodulation-cell division (RND) antibiotic efflux pump | rsmA | antibiotic efflux | + | + | |
| major facilitator superfamily (MFS) antibiotic efflux pump | emrB | antibiotic efflux | + | + | |
| major facilitator superfamily (MFS) antibiotic efflux pump | emrR | antibiotic efflux | + | + | |
| resistance-nodulation-cell division (RND) antibiotic efflux pump, General Bacterial Porin with reduced permeability to beta-lactams | marA | antibiotic efflux, reduced permeability to antibiotic | + | + | |
| major facilitator superfamily (MFS) antibiotic efflux pump, resistance-nodulation-cell division (RND) antibiotic efflux pump | H-NS | antibiotic efflux | + | + | |
| resistance-nodulation-cell division (RND) antibiotic efflux pump | baeR | antibiotic efflux | + | + | |
| ampC-type beta-lactamase | Escherichia coli ampH beta-lactamase | antibiotic inactivation | + | + | |
| fosfomycin thiol transferase | FosA2 | antibiotic inactivation | + | + | |
| ACT beta-lactamase | ACT-23 | antibiotic inactivation | + | + | |
| ATP-binding cassette (ABC) antibiotic efflux pump | msbA | antibiotic efflux | + | + | |
| Penicillin-binding protein mutations conferring resistance to beta-lactam antibiotics | Haemophilus influenzae PBP3 conferring resistance to beta-lactam antibiotics | antibiotic target alteration | + | + | |
| antibiotic-resistant UhpT | Escherichia coli UhpT with mutation conferring resistance to fosfomycin | antibiotic target alteration | + | + | |
| resistance-nodulation-cell division (RND) antibiotic efflux pump | Escherichia coli marR mutant conferring antibiotic resistance | antibiotic target alteration, antibiotic efflux | + | + | |
